# Editorial: Culture and emotion in educational dynamics

**DOI:** 10.3389/fpsyg.2024.1420573

**Published:** 2024-06-06

**Authors:** Enrique Riquelme, Silvia da Costa Dutra, Darío Páez

**Affiliations:** ^1^Education Faculty, Diversidad y Education Intercultural Department, Núcleo de Investigación en Estudios Interétnicos e Interculturales, Temuco Catholic University, Temuco, Chile; ^2^Faculty of Human and Social Sciences, Department of Psychology and Sociology, University of Zaragoza, Zaragoza, Spain; ^3^Facultad de Educacion y Ciencias Sociales, Andrés Bello University, Santiago, Chile

**Keywords:** culture, emotion, education, dynamics, students

Culture plays an important role in regulating the emotions and influencing the spread of education. Thus, the impact of culture on emotional dynamics in educational environments is a very important subject. It is particularly important in contexts of social and cultural diversity, where schools have to navigate through the cultural and emotional dynamics of the majority group.

The object of this area of investigation was to offer a global view of the current state of the art in the field of emotional dynamics in education, with a particular focus on how culture mediates these dynamics. This research seeks to integrate experiences and contributions from different regions of the world in order to offer full understanding of the role of culture in regulating the emotions in educational dynamics. In this editorial we offer a themed review of the fascinating and diverse contents of this Field of Investigation.

In the area of *Affectivity and education*, Frumos et al. examine how achievement emotions moderate relations between mastery and performance goal orientation and academic achievement in students. Self-efficacy proved to be the only significant mediator, and mastery avoidance goals were linked with high scores in motivational components at high levels of negative emotions. This approach offers a detailed view of the complexities of academic targets and their emotional connections.

Another area of investigation is Educational Management and Mental Health post-pandemic (Bonhomme and Rojas). This includes an analysis of how educational spaces have been transformed, as is shown by the proliferation of Social-Emotional Learning (SEL) policies in Chilean schools from the perspective of the (dis)continuities between institutions and personal effort, and between basic and continuous education. This manuscript highlights the diversity of approaches to research in education and mental health, from the practical implementation of policies to the exploration of emotions and the promotion of cultural competences in various educational contexts around the world. Students' experiences, whether in the integration of local culture into education or in the management of academic stress, underline the importance of holistic, culturally sensitive approaches to education and mental health.

The work of Chen shows us, from the perspective of a Chinese doctoral student, how personal growth experiences are developed. The thematic analysis revealed three recurrent themes: anxiety, enjoyment and personal growth. The results suggest the coexistence of anxiety and enjoyment, and fluctuation between them, during the doctoral research project.

The work of Jue and Kim on the relation between Art therapy students' burnout, practicum stress, and teacher support shows how practicum stress increases burnout while social support decreases it. The authors found particularly that professor support, rather than the support of colleagues or family, significantly reduced burnout. Likewise, they identified that academic support was more important than emotional support to reduce students' burnout.

Another area examined is the relation between Emotions and cultural identity. Based on a Web of Science analysis, Kuang et al. examine hotspots and frontiers of ethnic cultural identity. The investigation shows an increasing trend in this area, with universities in USA, UK, Australia and China leading exploration of subjects like emotional perception, multicultural identity processes and cultural adaptability. In the same line, Qian et al. investigate the Integration of the Shangshan Culture into STEAM education, stressing the importance of adding elements of Chinese culture, and of encouraging the application of interdisciplinary knowledge in artistic and creative exploration.

Ding et al. From Bronfenbrenner's bioecological model the study assesses the emotional, social, and physical wellbeing of Chinese migrant children in urban areas amid the COVID-19 pandemic, revealing significant disparities in academic advancement between migrant and local children, exacerbated by neglect from educators and policymakers, resulting in heightened anxiety, anger, and uncertainty about their future among migrant children. These educational inequalities underscore the urgent need for policy reform to address the disparities experienced by migrant children during the pandemic. Lawrie and Kim provide us an overview of a study concerning first-generation college students, emphasizing the significance of comprehending the psychological mechanisms behind their difficulties. It adopts a cultural psychology perspective and examines the impact of “emotional (mis)match” on the reduced wellbeing of these students. While emotional accuracy correlates with positive outcomes, it's notably lower among first-generation students. These findings stress the importance of grasping distinct emotional processes in the social adjustment of college students.

Finally, there is research into the relation of Extra-curricular and curricular activities with the emotions. The investigation carried out in Japan by Onoda and Omi highlights the value of extracurricular activities for secondary school students, focusing on the expression of increasing the attractiveness of school through writing. The consciousness of junior schoolmates increased the evaluation of extracurricular activities as attractions, stressing the importance of the transmission of values through writing.

In Australia, Pope et al. argues that the implementation of the Human Library as a teaching method resulted in a significant increase in the cultural competence of occupational therapy students. This flexible and economically attractive approach may be considered for developing competences in culturally congruent medical attention.

And Carrasco-Dajer et al. discusses the importance of digital literacy among older individuals to enhance their aging experience, highlighting that they face the largest digital gap. A study was conducted involving a digital literacy intervention for individuals aged 60 and above, with pre- and post-evaluations. The intervention resulted in significant improvements in digital literacy, with indirect effects observed on wellbeing, social support, and quality of life.

To summarize, this Research Topic highlights the importance of understanding the influence of culture on emotional dynamics in educational environments, especially in culturally diverse settings. Several studies were included examining emotional experiences in academic settings, the impact of the COVID-19 pandemic on migrant children's wellbeing, and the challenges faced by first-generation college students. Additionally, the Research Topic discusses the role of extracurricular activities and curricular interventions in shaping students' emotional experiences. Overall, these studies contribute valuable insights into the complex interplay between emotions, culture, and education, emphasizing the need for holistic and culturally sensitive approaches to promoting wellbeing and academic success.

## Author contributions

ER: Writing – original draft. SC: Writing – review & editing. DP: Writing – review & editing.

